# Barriers and facilitators of health professionals in adopting digital health-related tools for medication appropriateness: A systematic review

**DOI:** 10.1177/20552076231225133

**Published:** 2024-01-17

**Authors:** Daniela A. Rodrigues, Maria Roque, Ramona Mateos-Campos, Adolfo Figueiras, Maria Teresa Herdeiro, Fátima Roque

**Affiliations:** 1 Research Laboratory on Epidemiology and Population Health, Polytechnic of Guarda (IPG), Guarda, Portugal; 2 Health Sciences Research Centre, University of Beira Interior (CICS-UBI), Covilhã, Portugal; 3 16779 University of Salamanca, Salamanca, Spain; 4 Hospital de Santa Maria, Lisbon, Portugal; 5 Area of Preventive Medicine and Public Health, Department of Biomedical and Diagnostic Sciences, 16779 University of Salamanca, Salamanca, Spain; 6 Department of Preventive Medicine and Public Health, University of Santiago de Compostela, Santiago de Compostela, Spain; 7 Health Research Institute of Santiago de Compostela (IDIS), Santiago de Compostela, Spain; 8 Consortium for Biomedical Research in Epidemiology and Public Health (CIBER Epidemiology and Public Health-CIBERESP), Madrid, Spain; 9 Department of Medical Sciences, Institute of Biomedicine ( 451098 iBiMED), University of Aveiro, Aveiro, Portugal

**Keywords:** Systematic review, medication appropriateness, digital health tools, health professionals, barriers, facilitators

## Abstract

**Objective:**

Digital health is described as the use and development of all types of digital technologies to improve health outcomes. It could be used to prevent medication errors, a priority for health systems worldwide. However, the adoption of such tools remains slow. This study aims to identify factors (attitudes, knowledge and beliefs) acting as barriers and/or facilitators reported by healthcare professionals (HCPs) for the adoption of digital health-related tools for medication appropriateness.

**Methods:**

A systematic review was performed by searching the literature in the MEDLINE PubMed, and EMBASE scientific databases for original articles regarding qualitative and quantitative data.

**Results:**

Fifteen articles were included and a total of 125 barriers and 108 facilitators were identified, consolidated and categorized into technical (n = 48), organizational (n = 12), economical (n = 4), user-related (n = 34), and patient-related (n = 8) components. The most often reported barriers and facilitators were technical component-related ones concerning the need for additional training (n = 6), the time consumed (n = 6), and the easy way of using or learning how to use the tools (n = 9), respectively. Regarding setting analysis, agreement with clinical decision recommendations and impact on the doctor–patient relationship were more valued in primary care, while the user interface and system design were in the hospital.

**Conclusions:**

The barriers and facilitators identified in this study provide relevant information to developers and it can be used as a starting point for the designing of successful digital health-related tools, specifically related to medication appropriateness. Future research includes economic evaluation-focused studies and in-depth case studies of specific barriers and facilitators.

## Introduction

Digital health is understood as the use and development of all types of digital technologies to improve health outcomes,^1^ encompassing eHealth (information and communication technologies), mHealth (mobile health) and big data.^2^ The widespread use of health information technology has become one of the main strategies to improve the efficiency, accessibility, quality and safety of health systems, clinical decision making, and medication management, helping to avoid errors at ordering and prescribing stages.^3,4^ In fact, in one study, the likelihood of medication errors in hospitals decreased 48% when a prescription drug order was performed through a computerized provider order entry (CPOE) system.^5^

Nowadays, healthcare professionals (HCPs) and patients have access to many digital technology resources involving electronic health records (EHRs), electronic prescribing (ePrescribing), electronic medication administration records (eMARs), remote monitoring, telehealth, websites and applications available for computers, tablets, or smartphones, educational videogames, CPOE, clinical decision support systems (CDSS), bar code medication administration (BCMA), automated medication dispensing cabinets (ADCs) and patient data management systems (PDMSs) among others.^4,6–9^ Currently, more than 350,000 health applications are available, and 250 new ones are released every day.^10^ Data showed that 58.23% of smartphone users in the United States used and downloaded health-related applications.^11^ These tools could have several beneficial functionalities such as support for clinical diagnosis or decision making, patient adherence, or simply providing education.^12^ It is estimated that the digital health market should reach nearly 660 billion dollars by 2025.^13^ Previous studies found that the implementation of these technologies has been demonstrated to reduce costs by saving time and to improve patients’ health outcomes through a reduction of the occurrence of ADR caused by medication errors.^9,14–18^ In addition, the widespread of digital health could help to eliminate health disparities worldwide.^19^

Despite the advantages presented above, the adoption of digital health-related tools remains slow.^20,21^ As the digital era emerges, major changes in HCPs’ workflow arise, sometimes with a lack of regulation or guidelines, which has led to many HCPs remaining reluctant to adopt digital health technologies.^22,23^ Important knowledge gaps related to digital health education were also identified, reinforcing the need for wider support for HCPs.^24^ Previously, one study found that only five of 280 diabetes mobile applications evaluated were associated with clinically meaningful improvement, revealing that clinical effectiveness is not always guaranteed.^25^

Addressing factors that could hamper or promote the adoption of digital health-related tools can help to create technologies that better meet the needs of HCPs and provide patients with higher-quality care.^24,26^ Therefore, preferences of end-users should be considered for the technology design. HCPs will benefit from clinically meaningful digital solutions, while healthcare systems will need to obtain financial returns or save costs by acquiring digital health technologies.^27^ Therefore, to improve digital health adherence, an understanding of barriers and facilitators that can influence the adoption and use of digital tools by HCPs is the first step required.

Previous reviews on barriers and facilitators for the implementation of eHealth or mHealth in general,^21^ or the acceptance of medication-related CDSS,^28^ or in a specific setting^29,30^ showed the following as the most reported barriers and facilitators: time consuming,^28,29^ security and privacy concerns,^21,23,31^ limited knowledge and literacy,^21,31^ weakened communication and interaction with others,^23,29^ and financial support^21,23,31^ as barriers; ease of use,^21,23,28^ and equipment availability and reliability^21,28,29^ as facilitators. However, there is a lack of systematized information on the barriers and facilitators for the use of the various digital tools available to support the appropriate use of medicines. Challenges to the implementation of such technologies should be studied; therefore, with our review, we aim to expand previous findings by covering all types of digital health-related tools for medication appropriateness and to identify factors that are reported by HCPs to act as barriers and/or facilitators in the adoption of digital health-related tools for medication appropriateness. Therefore, outcomes from this systematic review will add more reliable information to the existing body of evidence for developers and all stakeholders involved in the design and creation of successful digital health-related tools.

## Material and methods

To provide a comprehensive understanding more useful for clinical decision making and health care decision makers, and to help researchers focus on the most relevant barriers and/or facilitators when developing any types of digital health-related tools, we performed a systematic review following the PRISMA (Preferred Reporting Items for Systematic Reviews and Meta-Analyses) 2020 guidelines (Table S1).^32^ The research protocol is registered on PROSPERO (CRD42022363235).

### Search strategy

A literature search was conducted on 28 October 2022 on the MEDLINE PubMed and EMBASE scientific databases for articles published since 1 January 2000. The search strategy intended to identify relevant studies addressing barriers and/or facilitators identified by HCPs in adopting digital health-related tools for medication appropriateness, using the following broad-based search terms strategy: “(barrier OR facilitator OR attitudes OR beliefs OR knowledge) AND (adopt OR adoption OR implementation*) AND (health professional OR health provider OR clinician OR physician OR GP OR general practitioner OR nurse OR pharmacist) AND (digital health-related tool OR digital health tool OR mobile health OR mhealth OR m-health OR electronic health OR ehealth OR e-health OR telehealth OR clinical decision support system OR computerized clinical decision support system) AND (medication appropriateness OR drug appropriateness OR appropriate medication OR inappropriate medication OR inadequate prescription OR quality prescription OR adequate prescription)” (Table S2). The reference list of relevant articles and the author’s files were also searched to identify potential additional articles (snowballing technique).

### Study selection

The following inclusion criteria were applied: (i) studies that identify factors (such as attitudes, knowledge, and/or beliefs) acting as barriers (factors hampering) or facilitators (factors promoting) in the adoption of digital health-related tools for medication appropriateness; (ii) studies directed to HCPs in any healthcare context (also including studies in which HCPs are a part of a heterogeneous sample when the data are presented separately for HCPs); (iii) studies that did not have investigating barriers and facilitators to be a mandatory aim; (iv) studies reporting the results of primary data; (v) studies published in Portuguese, English, or Spanish. For this review, an HCP was considered as any individual who requires a degree qualification to practise in their respective healthcare field and to provide healthcare treatments and advice based on formal training and experience.^33^

We excluded studies that do not present an analysis of barriers or facilitators as an outcome, reviews, meta-analyses, opinions, letters to the editor that do not provide original data, comments, reports, protocols, duplicate studies and the grey literature. We excluded usability studies, as these look more to analyse system-specific usability problems rather than barriers and facilitators. Studies that analyse electronic prescribing systems which do not address medication appropriateness were also excluded. Studies that identify barriers and facilitators but did not associate them with the intention of HCPs to adopt or use digital health-related tools were excluded.

Two researchers (DR and MR) independently screened all titles and abstracts retrieved from the databases according to the inclusion criteria and evaluated the eligibility of full-text articles. All discrepancies were resolved through discussion with the help of a third researcher (MTH, AF or FR).

### Outcome measures

Our primary outcome measure was the data establishing the specific barriers and/or facilitators reported by HCPs in adopting digital health-related tools for medication appropriateness.

### Quality assessment

The quality and susceptibility to bias were independently evaluated by two researchers (DR and MR) for each included study. The Mixed Methods Appraisal Tool (MMAT) version 2018 was used to assess the quality of qualitative, quantitative, and mixed methods studies.^34,35^ All discrepancies were resolved through discussion with a third researcher (MTH, AF or FR).

### Data extraction

Data were extracted from all studies by two authors (DR and MR) using a data extraction sheet. Data on authors (year), country, study design, setting, sample size, health professionals' category, methods of data collection, digital health tools, description of tools, types of treatments/drugs, and patients' subgroups were extracted from each article included. Further data about barriers and facilitators were extracted and analysed.

### Data analysis

We defined a barrier as a factor hampering the adoption of digital health-related tools and a facilitator as a factor promoting the adoption of digital health-related tools. Barriers and/or facilitators extracted from the included studies were independently analysed by two researchers, and grouped into the following categories: (i) technical, concerning all barriers and/or facilitators related to digital health tools itself and the associated technology and training; (ii) user-related, including all aspects related to the behaviours, concerns and feelings of HCPs; (iii) economical, aspects associated with costs, funding, financial incentives and/or reimbursement; (iv) organizational, including all barriers and/or facilitators related to the organizational structure and support in which digital health tools are integrated (or where it's supposed to be); (v) patient-related, including all concerns related to the patient and the quality of care provided. Discrepancies were resolved through discussion with the help of a third researcher. This analysis was conducted for each category concerning barriers and/or facilitators, regardless of the authors’ categorisation. The quotes transcribed in the included studies were also analysed. Subgroup analyses were conducted for the types of barriers and facilitators, and setting (hospital and primary care).

## Results

### Study selection

**Figure 1.**
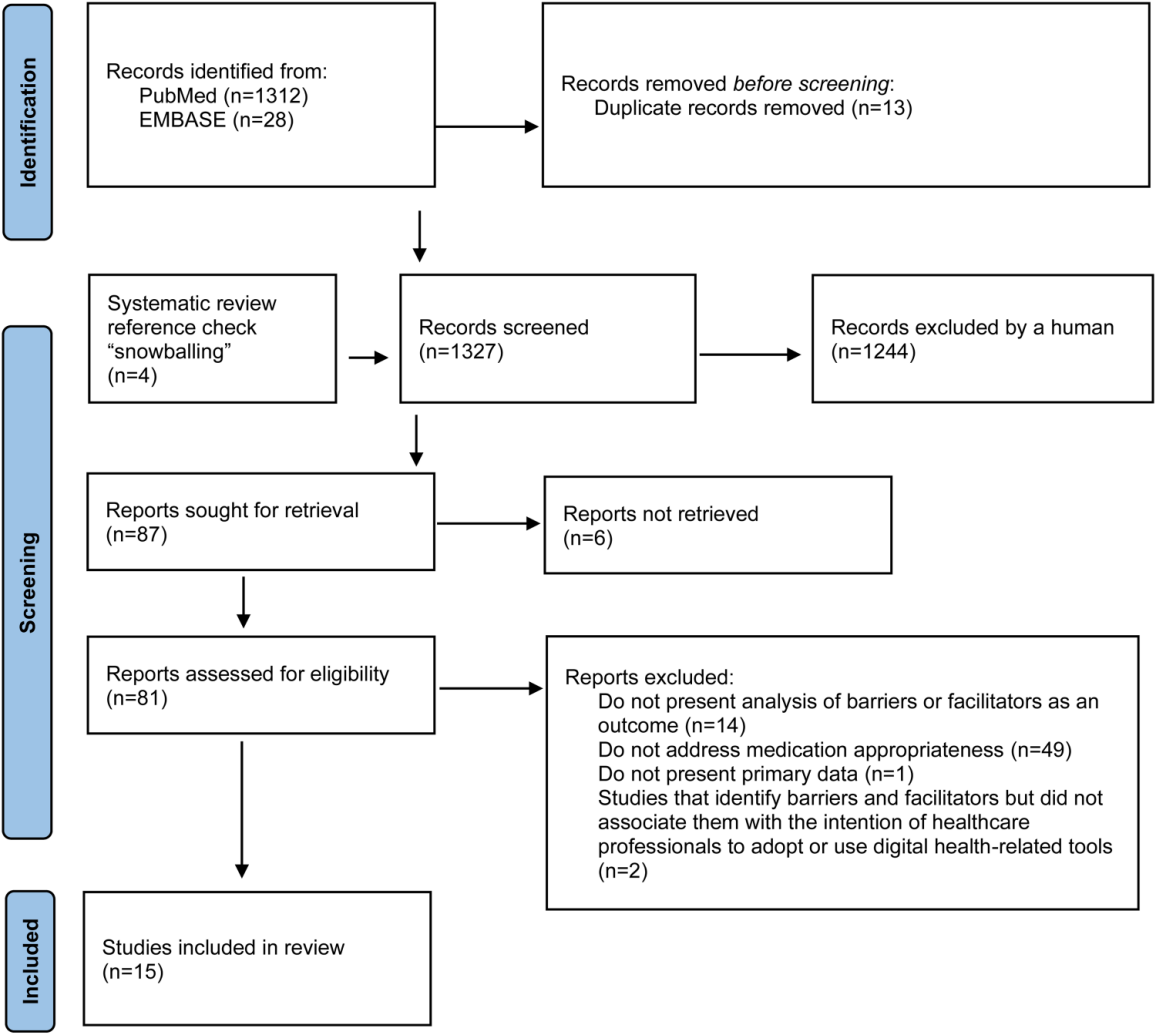
PRISMA diagram of the literature selection in this systematic review.

The search of the databases yielded 1340 citations by systematically searching the literature in the scientific databases MEDLINE PubMed (n = 1312) and EMBASE (n = 28). After screening titles and abstracts, 13 duplicated articles were removed, and four were added using the snowballing technique. A total of 1327 articles were screened and 87 potentially met the inclusion criteria. Since six articles were not retrieved, only 81 articles were fully screened. Among these articles, 66 were excluded because they did not present an analysis of barriers or facilitators as an outcome (n = 14), did not address medication appropriateness (n = 49), did not present primary data (n = 1), and did not associate barriers and/or facilitators with the intention of HCPs to adopt or use digital health-related tools (n = 2) (Table S3). As a result, 15 articles conducted in 11 countries fulfilled the inclusion criteria and they were included in this systematic review (Figure 1).^36–50^

### Studies characteristics

A general description of the characteristics of the included studies is presented in Table 1. Among them, nine were conducted in Europe,^36,37,39–42,46,48,50^ five in North America,^43–45,47,49^ and one in Asia.^38^ Most of the studies were qualitative (n = 9),^36,37,39–41,44,45,47,48^ followed by mixed methods studies (n = 4)^38,42,43,50^ and quantitative studies (n = 2).^46,49^ Regarding the setting, the most frequent was primary care (n = 7),^36,39–41,43,44,46^ followed by hospitals (n = 2)^37,38^ or both (n = 1),^48^ ambulatory care (n = 1),^47^ an academic internal medicine residency training clinic (n = 1),^49^ and a paediatric oncology centre (n = 1).^50^ In nine studies, the sample size was composed of a multidisciplinary team of several HCPs,^36,40–44,47,48,50^ three with physicians only,^37,38,49^ two with general practitioners (GPs)^39,46^ and one with nurses.^45^ The sample size ranged from 15 to 98 subjects. Four studies were conducted only through interviews^36,37,39,44^ and four through focus groups;^40,41,47,48^ two resorted to surveys,^46,49^ and the remaining five used a combination of methods.^38,42,43,45,50^ The most common type of digital health-related tool was CDSS (n = 11),^36–41,43–45,48,50^ followed by electronic prescribing systems (n = 2),^47,49^ CDSS with e-prescribing (n = 1),^46^ and a smartphone application (n = 1).^42^ Three studies were directed for antimicrobial drugs,^37,42,43^ one was for Potentially Inappropriate Medication (PIM),^39^ one for chemotherapy,^50^ one for insulin therapy^45^ and one for multiple diseases;^41^ the remaining eight studies were not directed to any specific pathology or treatment.^36,38,40,44,46–49^ In one study, the patient subgroup was older adults;^39^ in one study, it was children;^50^ in one study, it was multimorbid patients.^44^ Twelve studies were not targeted at any specific population.^36–38,40–43,45–49^

**Table 1.** Characteristics of the included studies (n = 15).

Author (year)	Country	Study design	Setting	Sample size	Health professionals’ category	Data collection method	Digital health tool	Description of tool	Type of treatment/drug	Patients’ subgroups	Quality assessment/(score obtained/total score)
Jeffries et al. (2021)^a^	England	Qualitative	Primary care	29	GP, N, Pharm, and PharmT	Interview	CDSS	Provided a suite of messages that appeared as alerts at the point of prescribing	Not directed to any specific pathology or treatment	Not targeted at any specific population	7/7
Catho et al. (2020)	Switzerland and France	Qualitative	Hospital	29	Phys	Interview	CDSS	Recommendations embedded into the EHR and the CPOE system	Antimicrobial	Not targeted at any specific population	7/7
Jung et al. (2020)	South Korea	Mixed methods	Hospital	61 (quantitative); 15 (qualitative)	Phys	Survey (quantitative); interview (qualitative)	Medication Decision Support System (CDSS)	MDSS in EHR with alert screens; prescription drug monitoring program	Not directed to any specific pathology or treatment	Not targeted at any specific population	15/17
Rieckert et al. (2018)	Germany	Qualitative	Primary care	21	GP	Interview	CDSS	CMR on the screen displaying recommendations regarding missing indications, necessary laboratory tests, evidence-based current medication, dose adjustments, potentially harmful drug-drug interactions, contra-indications, and possible adverse drug events	Several guidelines were used to access medication appropriateness	Older patients (aged 75 and older) taking at least 8 drugs	7/7
Koskela et al. (2016)	Finland	Qualitative	Primary care	21	Phys (9) and N (12)	Focus groups	CDSS	Evidence-based MEDS that provides patient-specific clinical recommendations or warnings in the form of reminders and links to guidelines. Contains CMR where is available guidance for an individual patient on drug therapy	Not directed to any specific pathology or treatment	Not targeted at any specific population	7/7
Lugtenberg et al. (2015)	Netherlands	Qualitative	Primary care	24	GP and N	Focus groups	CDSS	It provides evidence-based patient-specific advice during consultation in terms of patient data registration, drug prescription and management; in case of a discrepancy between current (guidelines) and advised care, an alert is sent	Cardiovascular risk management, Asthma/COPD, Diabetes mellitus type II, Thyroid disorders, Viral hepatitis and other liver diseases, Atrial fibrillation and Subfertility, Gastro protection and Chronic renal failure	Not targeted at any specific population	7/7
Charani et al. (2013)	UK	Mixed methods	Hospital	81 (quantitative); not reported (qualitative)	Physc and Pharm	Survey (quantitative); Focus groups (qualitative)	Smartphone application	Therapeutic drug monitoring and clinical calculators	Antibiotic	Not targeted at any specific population	13/17
Litvin et al. (2012)	USA	Mixed methods	Primary care	39	Phys (33) and N (6)	Data extracted from the EHR (quantitative); Interview (qualitative)	CDSS	Diagnostic criteria; prompts regarding appropriate antibiotic use; first-line antibiotics recommendations; educational material	Antibiotic for acute respiratory infections	Not targeted at any specific population	14/17
Vedel et al. (2012)	Canada	Qualitative	Primary care	31	Phys, N, and Pharm	Interview	CDSS	Traditional EMR functionalities plus advanced features, such as decision-support tools, patient-centred goal setting, and interdisciplinary functionalities	Not directed to any specific pathology or treatment	Multimorbid patients	7/7
Campion et al. (2011)	USA	Qualitative	Hospital	37	N	Direct observation and interviews	CDSS	Calculates insulin rates using a linear equation explaining the recommendations	Intensive insulin therapy in the surgical and trauma intensive care units	Not targeted at any specific population	5/7
Hor et al. (2010)	Ireland	Cross-sectional (quantitative)	Primary care	98	GP	Survey	CDSS within electronic prescribing	An algorithm that establishes the safety and appropriateness of a prescription, with links to an information system employed to enhance the safety of the prescription. This may include clinical checks which allow alerts to be flagged up to the prescribers on drug-drug interactions or formulary status	Not directed to any specific pathology or treatment	Not targeted at any specific population	6/7
Weingart et al. (2009)	USA	Qualitative	Ambulatory care	25	Phys (21), N (3), and PhysA (1)	Focus groups	Electronic prescribing system	A linked desktop and handheld electronic prescribing system with a variety of drug allergy and interaction alerts, medication pick lists with default dosing, the ability to create favourite prescriptions, formulary tiers tied to patient's insurance type, and the ability to transmit prescriptions to the pharmacy electronically	Not directed to any specific pathology or treatment	Not targeted at any specific population	7/7
Varonen et al. (2008)	Finland	Qualitative	Primary and secondary care	39	Phys (22 primary care, 17 sary care)	Focus groups	CDSS	Evidence-based MEDS with several updated guidelines	Not directed to any specific pathology or treatment	Not targeted at any specific population	6/7
Schectman et al. (2005)	USA	Not reported (quantitative)	Academic internal medicine residency training clinic	84	Phys	Survey	Computer-based prescription expert system (electronic prescribing system)	Enables electronic maintenance of a medication list, printing and renewing prescriptions, and checks drug-drug and drug-allergy interactions	Not directed to any specific pathology or treatment	Not targeted at any specific population	6/7
Bury et al. (2004)	UK	Mixed methods	Pediatric Oncology Centres and Pediatric Oncology Shared Care Units	36	Clinicians	Survey (quantitative); Interview (qualitative)	Web-based decision support system (CDSS)	A shared electronic record of treatment during continuing therapy, making haematology results, dosage history and other clinical data accessible; provides protocol-based decision support for clinicians when modifying doses of oral chemotherapy – the system advises on any modifications to the level of both oral chemotherapeutic agents for that week and presents an explanation for its proposal	Chemotherapy for Acute Lymphoblastic Leukemia	Children	9/17

CDSS: clinical decision support system; CMR: comprehensive medication review; COPD: chronic obstructive pulmonary disease; CPOE: computerized physician order entry; EHR: electronic health record; EMR: electronic medical record; GP: general practitioner; HCP: healthcare professionals; MDSS: medication decision support system; MEDS: medicine electronic decision support; N: nurse; Pharm: pharmacist; PharmT: pharmacy technicians; Phys: physician; PhysA: physician assistant; UK: United Kingdom; USA: United States of America

^a^ Studies in which HCPs are a part of a heterogeneous sample but it is possible to distinguish their concrete opinions; the sample size reported corresponds only to HCPs.

### Quality assessment

The quality assessment result of each study is reported in Table 2. Twelve articles fulfilled more than 80% of the exploratory questions.^36–41,43,44,46–49^ Regarding qualitative studies, the main limitation was related to the fact that sometimes the results are not sufficiently substantiated by the data. In quantitative studies, the sampling strategy and the representativeness of the target population were the major limitations.

**Table 2.** Quality assessment of the included studies through the mixed methods appraisal tool (MMAT) version 2018.

Mixed Methods Appraisal Tool (MMAT) for qualitative studies				
No	Methodological quality criteria	Number of studies (n = 13)		
		Yes	No	Can’t tell
1.1	Is the qualitative approach appropriate to answer the research question?	13	0	0
1.2	Are the qualitative data collection methods adequate to address the research question?	12	1	0
1.3	Are the findings adequately derived from the data?	13	0	0
1.4	Is the interpretation of results sufficiently substantiated by data?	10	3	0
1.5	Is there coherence between qualitative data sources, collection, analysis and interpretation?	13	0	0
Mixed Methods Appraisal Tool (MMAT) for quantitative descriptive studies				
No	Methodological quality criteria	Number of studies (n = 6)		
		Yes	No	Can’t tell
4.1	Is the sampling strategy relevant to address the research question?	1	5	0
4.2	Is the sample representative of the target population?	3	3	0
4.3	Are the measurements appropriate?	4	2	0
4.4	Is the risk of nonresponse bias low?	4	2	0
4.5	Is the statistical analysis appropriate to answer the research question?	5	1	0
Mixed Methods Appraisal Tool (MMAT) for mixed methods studies				
No	Methodological quality criteria	Number of studies (n = 4)		
		Yes	No	Can’t tell
5.1	Is there an adequate rationale for using a mixed methods design to address the research question?	2	2	0
5.2	Are the different components of the study effectively integrated to answer the research question?	4	0	0
5.3	Are the outputs of the integration of qualitative and quantitative components adequately interpreted?	3	1	0
5.4	Are divergences and inconsistencies between quantitative and qualitative results adequately addressed?	4	0	0
5.5	Do the different components of the study adhere to the quality criteria of each tradition of the methods involved?	2	2	0

### Barriers and facilitators related to the use of digital tools on medication appropriateness

In the 15 included studies, 125 barriers and 108 facilitators were identified. After they were consolidated, 59 unique different types of barriers (Table 3) and 47 unique different types of facilitators (Table 4) were obtained. Among the barriers, 24 were classified as technical, 22 were user-related, one was considered economical, nine were organizational, and three were patient-related. Regarding facilitators, 24 were of the technical category, 12 were user-related, three were considered economical, three were organizational, and five were patient-related. Barriers and facilitators according to each study can be found in Table S4.

**Table 3.** Classification (and acronyms) of barriers.

Classification	Acronym	Total studies (n)	Qualitative studies (n)	Quantitative studies (n)	Mixed methods studies (n)	References
*Technical barriers*		*14*	*9*	*2*	*3*	
Alert fatigue	AF	4	4	0	0	^36,37,41,47^
Bothersome to use	BTU	1	0	0	1	^38^
Ensure data security	EDS	1	1	0	0	^39^
Hard to use	HU	1	0	0	1	^38^
Hardware problems	HP	1	1	0	0	^47^
Lack of acceptable, standardized product software	LASPS	1	0	1	0	^46^
Lack of accuracy	LA	1	0	0	1	^38^
Lack of connection with other medical software	LCOMS	2	2	0	0	^39,41^
Lack of customizability	LC	2	1	0	1	^38,41^
Lack of learning capacity of the system	LLCS	1	1	0	0	^41^
Lack of prioritization of reminders	LPOR	1	1	0	0	^40^
Lack of protocol reminders	LPR	1	1	0	0	^45^
Limited value of the alerts	LVA	4	3	0	1	^40,41,43,48^
The low specificity of alerts	LSA	2	2	0	0	^41,47^
Need for training	NT	6	2	1	3	^36,38,41,43,49,50^
Not considering suspended/paused medications	NCSPM	1	1	0	0	^40^
Not consistent in terms of usability	NCTU	1	0	0	1	^38^
Not well-integrated functions in the system	NIFS	2	1	0	1	^47,50^
Outdated medication information	OMI	1	1	0	0	^40^
Poor user interface and system design	PUISD	5	4	0	1	^38,40,41,45,48^
Slow the clinical system down	SCSD	1	1	0	0	^36^
Time-consuming	TC	6	6	0	0	^37,39–41,44,45^
Unreliable transmission of information	UTI	1	1	0	0	^47^
Unusual to use	UTU	1	1	0	0	^39^
*Organizational barriers*		*9*	*7*	*1*	*1*	
Alters workflow	AW	1	0	0	1	^43^
Double documentation	DD	1	1	0	0	^45^
Lack of a strategic plan for implementation	LSPI	1	0	1	0	^46^
Lack of common instructions in the organization	LCIO	1	1	0	0	^40^
Lack of internet	LI	3	1	0	2	^39,42,43^
Limited access to computers and other devices	LACOD	3	0	0	3	^42,43,50^
Medico-legal issues and liability	MLIL	3	2	0	1	^37,45,50^
System sustainability	SST	1	1	0	0	^44^
Use smartphone devices at work	USDW	1	0	0	1	^42^
*Economical barriers*		*5*	*3*	*1*	*1*	
Lack of funding and/or financial incentives	LFFI	5	3	1	1	^36,39,46,47,50^
*User-related barriers*		*14*	*9*	*2*	*3*	
Deters residents from learning the evidence-based guidelines	DRLEBG	1	1	0	0	^44^
Difficulties navigating the system	DNS	2	1	0	1	^47,50^
Disagreement with the clinical decision recommendations	DCDR	2	1	0	1	^39,43^
End-user unfamiliarity with the device and technology	EUUDT	4	3	0	1	^37,40,42,44^
Fear of misuse of data	FMD	1	1	0	0	^39^
Forgetting or delay in changing medication	FDCM	1	1	0	0	^39^
Flexibility to override the system	FOS	1	0	1	0	^46^
Lack of comfort using the system	LCUS	1	1	0	0	^44^
Lack of time	LT	3	3	0	0	^39,41,47^
Lack of trust in the system	LTS	3	2	1	0	^39,41,46^
Less rigorous consideration for individual patients	LRCIP	1	0	0	1	^50^
Long time to get used	LTGU	1	0	0	1	^38^
Loss of clinical autonomy	LCA	3	2	1	0	^37,46,48^
Negative impact on the doctor–patient relationship	NIDPR	4	4	0	0	^36,41,44,48^
No perceived usefulness	NPU	1	1	0	0	^44^
Only doctors can access the system	ODCAS	1	1	0	0	^40^
Preference for hand-written prescriptions	PFHWP	1	0	1	0	^49^
Pressure to follow the protocol strictly	PFPS	1	0	0	1	^50^
Previous experience with imperfect/dysfunctional systems	PEIDS	1	1	0	0	^48^
Reluctance to change	RC	3	2	0	1	^37,43,48^
Reluctance to discontinue medication prescribed by other specialists	RDMPOS	1	1	0	0	^39^
Use smartphone devices in the presence of patients	USDPP	1	0	0	1	^42^
*Patient-related barriers*		*2*	*1*	*0*	*1*	
Medication priorities of the patient	MPP	1	1	0	0	^39^
Patient cooperation in changing medication	PCCM	1	1	0	0	^39^
Patients with multiple complaints	PMC	1	0	0	1	^43^

**Table 4.** Classification (and acronyms) of facilitators.

Classification	Acronym	Total studies (n)	Qualitative studies (n)	Quantitative studies (n)	Mixed methods studies (n)	References
*Technical facilitators*		*13*	*7*	*2*	*4*	
Active profile management	APM	1	1	0	0	^36^
Beneficial calculators	BC	1	1	0	0	^40^
Easy access to up-to-date information	EAUTDI	2	2	0	0	^36,37^
Ease of use and learning how to use	EULHU	9	6	1	2	^36–38,40,44,47–50^
Educational role	ER	3	2	0	1	^36,47,50^
Enhance medication safety	EMS	2	2	0	0	^36,40^
The flexibility of the system	FS	1	1	0	0	^48^
Free of commercial advertisement	FCA	1	1	0	0	^39^
High sensitivity	HS	1	0	1	0	^46^
Improve prescribing quality	IPQ	1	0	1	0	^46^
Independent from the pharmaceutical industry	IPI	1	1	0	0	^39^
Integrates functions well	IFW	1	1	0	1	^38^
Pleasant presentation of data	PPD	2	1	0	1	^47,50^
Practice support	PS	5	4	1	0	^36,39,40,47,49^
Reduction in medication errors	RME	3	2	1	0	^37,46,47^
A reliable base of knowledge	RBK	1	1	0	0	^48^
Simplifies the prescription renewal process	SPRP	1	1	0	0	^47^
Staff members able to troubleshoot system problems	SMATSP	1	0	0	1	^43^
System security	SS	1	0	0	1	^50^
Time-saving and fast	TSF	5	3	1	1	^36,37,39,43,49^
Training provided	TP	3	2	0	1	^37,43,48^
Update and complement knowledge	UCK	2	1	0	1	^39,42^
Useful lab monitoring alerts	ULMA	1	1	0	0	^40^
Useful reminders	UR	4	4	0	0	^39,40,47,48^
*Organizational facilitators*		*2*	*0*	*1*	*1*	
Possibility to accommodate practice workflow	PAPW	1	0	0	1	^43^
Non-medical clinical staff having access to the system	NMCSAS	1	0	0	1	^43^
Willingness to invest greater resources in the future	WIGRF	1	0	1	0	^46^
*Economical facilitators*		*4*	*4*	*0*	*0*	
Financial incentives	FI	1	1	0	0	^47^
Adequate budgeting	AB	1	1	0	0	^48^
Improve cost effectiveness	ICE	2	2	0	0	^36,37^
*User-related facilitators*		*12*	*6*	*2*	*4*	
Agreement with the clinical decision recommendations	ACDR	2	1	0	1	^39,43^
Comfort in using it	CFU	1	1	0	0	^44^
Commitment to use it	CU	4	0	2	2	^38,43,46,49^
Encourages health professionals to reflect on their practice	EHPRP	3	2	0	1	^36,39,50^
Familiarity with the system and technology	FST	2	1	1	0	^44,46^
Improve adherence to policies	IAP	1	0	0	1	^42^
Improve communication	IC	2	1	0	1	^47,50^
Increases awareness of prescribing guidelines	IAPG	1	0	0	1	^43^
Increases clinician confidence	ICC	1	1	0	0	^39^
Matches expectations	ME	2	1	1	0	^44,49^
Societal trend	ST	1	1	0	0	^44^
Trust in the system	TS	6	4	0	2	^36–39,45,50^
*Patient-related facilitators*		*4*	*2*	*1*	*1*	
Enhance patient medication safety	EPMS	1	1	0	0	^39^
Improve communication with the patient	ICP	1	0	0	1	^43^
Improve patient education	IPE	1	0	0	1	^43^
Improve quality of care provided	IQC	2	1	1	0	^44,49^
Patient approval	PA	1	0	1	0	^49^

#### Technical barriers and facilitators

The most reported technical barriers were the need for training (n = 6)^36,38,41,43,49,50^ and time consumed (n = 6),^37,39–41,44,45^ closely followed by the poor user interface and system design (n = 5),^38,40,41,45,48^ alert fatigue (n = 4),^36,37,41,47^ and the limited value of the alerts (n = 4).^40,41,43,48^ Less reported ones were the lack of connection with other medical software (n = 2),^39,41^ the lack of customizability (n = 2),^38,41^ the low specificity of the alerts (n = 2),^41,47^ and the lack of well-integrated functions in the system (n = 2).^47,50^ The remaining technical barriers were reported only one time.

The most cited technical facilitators were the ease of use and learning how to use (n = 9),^36–38,40,44,47–50^ the support given to clinicians (n = 5),^36,39,40,47,49^ time saved (n = 5),^36,37,39,43,49^ and useful reminders (n = 4).^39,40,47,48^ Next, the educational role (n = 3),^36,47,50^ reduction in medication errors (n = 3),^37,46,47^ and the training provided (n = 3) were cited.^37,43,48^ The least described ones were the easy access to up-to-date information (n = 2),^36,37^ the possibility to enhance medication safety (n = 2),^36,40^ the pleasant presentation of data (n = 2),^47,50^ and the opportunity to update and complement knowledge (n = 2).^39,42^ The remaining ones were reported only one time.

#### User-related barriers and facilitators

Regarding user-related barriers, the most reported were end-user unfamiliarity with the device and technology (n = 4),^37,40,42,44^ the negative impact on the doctor–patient relationship (n = 4),^36,41,44,48^ the lack of time (n = 3),^39,41,47^ the lack of trust in the system (n = 3),^39,41,46^ the loss of clinical autonomy (n = 3)^37,46,48^ and the reluctance to change (n = 3).^37,43,48^ Less reported ones were the difficulties navigating in the system (n = 2)^47,50^ and the disagreement with the clinical decision recommendations (n = 2).^39,43^ The remaining barriers were reported only one time.

The most reported user-related facilitators were the trust in the system (n = 6),^36–39,45,50^ the commitment to use it (n = 4)^38,43,46,49^ and the opportunity for HCPs to reflect it in their practice (n = 3).^36,39,50^ The less reported ones were the agreement with the clinical decision recommendations (n = 2),^39,43^ the familiarity with the system and the technology (n = 2),^44,46^ the improvement of communication between HCPs (n = 2),^47,50^ and the system meeting expectations (n = 2).^44,49^ The remaining user-related facilitators were reported only one time.

#### Economic barriers and facilitators

Only one economic barrier was reported concerning the lack of funding and/or financial incentives (n = 5).^36,39,46,47,50^

Regarding economic facilitators, the most cited was the improvement of cost-effectiveness (n = 2).^36,37^ Financial incentives (n = 1)^47^ and adequate budgeting (n = 1)^48^ were the least reported ones.

#### Organizational barriers and facilitators

The most reported organizational barriers were the lack of internet (n = 3),^39,42,43^ the limited access to computers and other devices (n = 3),^42,43,50^ and medico-legal issues and liability (n = 3).^37,45,50^ The remaining ones were only cited once.

Only three organizational facilitators were reported, the possibility to accommodate practice workflow (n = 1),^43^ the possibility of non-medical staff having access to the system (n = 1),^43^ and the willingness to invest greater resources in the future (n = 1).^46^

#### Patient-related barriers and facilitators

Three patient-related barriers were described, the willingness to change according to the medication priorities of the patient (n = 1),^39^ patients’ cooperation in changing medication (n = 1),^39^ and the inconvenience to use the system for patients with multiple complaints (n = 1).^43^

The most cited patient-related facilitator was the improved quality of care provided to patients (n = 2).^44,49^ The remaining ones were only reported once.

### Setting analysis

Alert fatigue, the time consumed, and the ease of use were common technical barriers and facilitators among primary care^36,39–41,44^ and hospitals^37,38,45^ (Table S5). Trust in the system was the common user-related facilitator.^36–39,45^

However, some differences were also found. Regarding the technical component, HCPs valued the need for training (n = 3) in primary care^36,41,43^ and the poor user interface and system design (n = 2) was more outlined in hospitals.^38,45^ While in primary care, the lack of internet was an organizational barrier (n = 2),^39,43^ medico-legal issues and liability were most reported in hospitals (n = 2).^37,45^ Negative impact on the doctor–patient relationship (n = 3) and lack of trust in the system (n = 3) were user-related barriers in primary care,^36,39,41,44,46^ while in hospitals, HCPs gave more importance to the end-user unfamiliarity with the device and technology (n = 2).^37,42^ Finally, besides the trust in the system, HCPs also reported the encouragement to reflect on their practice (n = 2),^36,39^ the agreement with the clinical decision recommendations (n = 2)^39,43^ and the commitment to use the system (n = 2)^43,46^ as user-related facilitators in primary care for the adoption of digital health-related tools.

## Discussion

Our review revealed that there are more barriers than facilitators in the adoption of digital health-related tools for medication appropriateness, suggesting that there is a lot of work to be done. The included studies mostly reported barriers and facilitators related to the technical component of the tool, more specifically, the need for additional training and the time consumed as barriers, and the easy way of using or learning how to use it as a facilitator.

HCPs do not want to waste time that could be spent with patients during the consultation with a digital tool that requires too much additional work rather than simplifying it,^41,51^ possibly lowering the quality of care provided.^37^ Otherwise, there is a propensity for simple systems that require minimal effort to use, view and understand, with an easy way to navigate through them.^36–38,40,44,47–50^ To overcome the need for training and the time needed for technology adoptation, user-centred approaches concerning literacy needs, skills, and capabilities should be developed and incorporated into the design of digital health technologies.^52^

Aligned with our findings, a previous systematic review about barriers and facilitators for the acceptance of medication-related CDSS identified the time consumed as the most reported barrier, and the ease of use as the most reported facilitator.^28^ Similar results were found in one study with a specific focus on digital health technology adoption for hypertension management^53^ and in EHR adoption by physicians.^51^ User-friendliness was also reported as one of the most important factors identified by patients and clinicians about digital technology regarding cardiovascular care,^54^ and as the most important predictor of the implementation of eHealth services and digital health technologies.^21,55^ Our results corroborate with a recently published overview of systematic reviews, which found that infrastructure and technical issues are a source of concern for HCPs in utilizing digital health technologies.^56^ To surpass such constrains, digital tool developers should guarantee that user interfaces are intuitive, and technical support is properly supplied.^57^ Investment in infrastructure upgrades may also be necessary to improve equipment and network connections.

Regarding user-related barriers and facilitators, end-user unfamiliarity with the device and technology was reported as a barrier to the adoption of new technologies. Reluctance to change and the lack of trust in the system were also important barriers reported by HCPs since they may be accustomed to traditional methods, resisting to embrace digital technology.^58^ User-friendly interfaces and well-designed digital tools that guarantee long-term sustainability and maintenance are essential for HCPs to integrate these tools into their daily practice, even without extensive technical knowledge.^19,27^ Besides, as a facilitator, the recommendations provided by these technologies can represent an opportunity for HCPs to reflect them in their practice and to consider alternative treatment options, leading to more informed decision making and making their practice even more patient-centred.^59^

The improved quality of care provided to the patient was also reported as one patient-related facilitator. On the other hand, inconvenience to use such tools in patients with multiple complaints was reported as one patient-related barrier, maybe due to the complexity of their health conditions and the need for a more comprehensive assessment and care plan.^60^ Therefore, a digital tool must be flexible. Sometimes patients can also resist to medication changes; however, digital health technologies could help to improve treatment adherence and completion.^61^ Reaching patient cooperation requires effective communication and education by HCPs.

Concerning the organizational domain, medico-legal issues and liability were reported as barriers. This could be related with misdiagnosis or errors such as providing inaccurate information, diagnostic suggestions, or treatment recommendations which could lead to patient harm, implying that digital tools may face liability claims.^62^ Otherwise, the growing recognition of significant benefits that technology can bring to healthcare increases the willingness to invest greater resources in digital health-related tools.^58^

The barriers and facilitators identified in our review aligned with existing models and frameworks theorizing user adoption of new technologies including: the Theory of Planned Behaviour (TPB),^63,64^ in which perceived behavioural is determined by the perceived availability of resources, opportunities and skills (like user unfamiliarity with the device and technology, or difficulties navigating through the system); the Theory of Interpersonal Behaviour (TIB)^64,65^ and the Social Cognitive Theory (SCT),^64,66^ with emotions, social and environment factors and habits being identified as the main factors influencing the intention to use (such as societal trend and the increase in clinicians’ own confidence); the Technology Acceptance Model (TAM),^64,67^ reflected in the perceived ease of use and attitude toward use; the Motivational Model (MM),^64,68^ being the improvement of the quality of care provided a motivation factor for the adoption of the digital tool for medication appropriateness.

Regarding the setting, most differences between primary care and hospitals are related to the technical component of the digital tool and user-related barriers and facilitators. In primary care, medication-related digital tools provide information to HCPs at the point of care for decision making and direct intervention.^69^ Therefore, agreement with the clinical decision recommendations and the impact on the doctor–patient relationship are very important factors.^70^ Besides, the relationship between HCPs and patients is one of the main factors influencing the delivery of good therapy and high-quality care.^71^ In hospitals, medication-related digital technology is directed towards therapeutic drug monitoring and clinical calculators,^42,45^ explaining why user interfaces and system design were more valued. A previous systematic review focused on hospital setting identified hardware and network problems, such as malfunctions and cumbersome access procedures, slow systems, and poor functionality as key barriers for the implementation of electronic systems for the prescribing, dispensing and administration of medicines.^29^ Also, it had already been reported that HCPs working in hospitals have the perception that CDSS may be used against them in the event of medico-legal controversies.^72^ Clear documentation and legal protection when using digital tools is advised.

The studies included in this review are reported in different years, with up to 20 year of difference, and it is possible to perceive that some barriers and facilitators remain over time. Although there is a recommendation from the United Nations^73,74^ and World Health Organization (WHO)^1^ for health digitalization, this study demonstrates that the lack of funding and financial incentives continues to be an important barrier that has not yet been overcome,^36,50^ as also reported by other studies.^75–77^ Funding challenges could be related to the cost of purchasing the system, expensive equipment, installation charges and staff training costs, among others.^36,39,46,47,50,76^ Unfortunately, an insufficient level of sustainable funding is an enormous obstacle to implementing digital health technologies.^21,78,79^ Knowledge about digital health outcomes is essential to overcome this barrier,^1^ with designing and planning for longitudinal cost analysis at the outset.^80^ Therefore, investment should be directed towards reducing these barriers and enhancing the facilitators when developing tools and with interventions adapted to cultural and sociodemographic differences. Regarding facilitators, HCPs continue to give the same importance to the ease of use of systems, the capacity to trust in digital tools, and the encouragement to reflect on their practice.^36,50^ Having a system that easily adapts to the needs and capabilities of HCPs is a timeless quality. Trust in digital health technologies can be positively influenced by a variety of factors, such as the system's capacity to provide the information needed at the point of prescribing, the ability to provide accurate messages, and the credible source of recommendations available in the system.^36–39,45,50,81^ Digital health systems can be a source of information that could support reflective practice and behaviour change of HCPs,^82^ and make the decision to prescribe a more conscious process.^36,39,50^ This is closely related to the educational role technical facilitator also identified in this study, since the alert information provided may educate the HCP about a potential interaction or prompt him/her to look up more information or confer with a colleague.^36,47,50^

Over the last 20 years, most of the studies were conducted in Europe, showing that the impact of digital tools for medication appropriateness on health was not well documented, especially in African countries. Despite reports of the use of mobile phones, and information and communication technologies to improve public health in developing countries,^83^ its application remains slow and inequitable, far from being high.^84^ This could be related to the lack of HCPs in these countries, the high cost of technology implementation, unstable power supply, the excessive burden of diseases^85^ or the limited access to digital technologies.^85,86^ However, according to the WHO Global Strategy on Digital Health, there is an urgent need to invest and overcome the major obstacles felt by developing countries in engaging and accessing digital health technologies.^1^ Therefore, a commitment and engagement with stakeholders are needed to advance digital health in these countries.

The data of this study also suggested that CDSS are the most used type of digital health-related tool for medication appropriateness, often making use of web applications, and in EHR or CPOE systems, maybe because several studies have shown that the use of these systems decreased the incidence of medical errors, improving the quality of care provided and patient safety.^80,87–89^ The high use of CDSS can also be explained by its capacity to develop multiple functions beyond medication reconciliation, such as computerized guidelines, order sets, patient data reports, documentation templates, and clinical workflow tools.^80^ They can also be found in different ways such as desktops, tablets, smartphones, biometric monitoring, and wearable health technology. Furthermore, CDSS can be cost-effective for health systems.^80^

With this study, we intend to give relevant information to developers and to all stakeholders for the creation and implementation of digital health-related tools for medication appropriateness. The strengths of this review also include the cross-checking of the reference lists of the included studies to avoid missing papers, and the independent and duplicate process of the screening, data extraction, and quality assessment. Besides, we made a reflection on differences regarding types of barriers and facilitators, and between settings. However, some limitations are also present. Firstly, a search in grey literature was not performed. However, we believe that it does not influence our results since the grey literature is not peer reviewed and is not indexed in major bibliographic resources. Secondly, the search strategy was limited to articles written only in English, Portuguese, and Spanish. Besides, the included studies were heterogeneous concerning digital health-related tools, although most studies used CDSS; the description can be variable in each study. Furthermore, medication appropriateness is generally understood as a guarantee that a specific medication is chosen as the most suitable, effective, and safety option aligned with the individual patient's specific clinical and healthcare needs, minimizing adverse effects and improving therapeutic outcomes.^90,91^ However, authors of the different studies included in this systematic review may have a different definition.

Despite the limitations presented, we believe that our main findings are pertinent. The data collected assessed the barriers and facilitations identified by HCPs for the adoption of medication-related digital tools, adding reliable information for all stakeholders, from developers to policymakers, allowing them to develop and implement useful and friendly health technologies, and to contribute to the improvement of services and healthcare.

## Conclusion

The barriers and facilitators identified in this study can be used as a starting point by developers and all stakeholders involved in the design and creation of successful digital health-related tools. The results suggest that the main barriers are related to usability and technical issues, user attitudes and perceptions, and infrastructure challenges; adherence to digital tools can be facilitated by practice support, user-friendly design and functionality, and quality improvement. The implementation of such tools should mostly follow a technical and user-centred approach, being easy-to-use essentially for HCPs without being time-consuming.

Future research is needed on economic evaluation-focused studies to surpass barriers and to better invest resources in medication-related digital tool development and implementation. In-depth case studies of specific barriers and facilitators should also be conducted.

## Contributorship

Research idea was conceived and conceptualization was performed by Daniela A. Rodrigues, Maria Roque, Ramona Mateos-Campos, Adolfo Figueiras Guzmán, Maria Teresa Herdeiro and Fátima Roque. Material preparation, data collection and analysis were performed by Daniela A. Rodrigues and Maria Roque. The first draft of the manuscript was written by Daniela A. Rodrigues. All authors reviewed, edited, and commented on previous versions of the manuscript. All authors read and approved the final manuscript.

## Supplemental Material

sj-docx-1-dhj-10.1177_20552076231225133 - Supplemental material for Barriers and facilitators of health professionals in adopting digital health-related tools for medication appropriateness: A systematic review Click here for additional data file. Supplemental material, sj-docx-1-dhj-10.1177_20552076231225133 for Barriers and facilitators of health professionals in adopting digital health-related tools for medication appropriateness: A systematic review by Daniela A. Rodrigues, Maria Roque, Ramona Mateos-Campos, Adolfo Figueiras, Maria Teresa Herdeiro and Fátima Roque in DIGITAL HEALTH
