# Supplementary material for: Barriers and facilitators of health professionals in adopting digital health-related tools for medication appropriateness: A systematic review
Source: Digit Health. 2024 Jan 17;10:20552076231225133. doi: 10.1177/20552076231225133 (PMC10798074; doi:10.1177/20552076231225133)
Supplement: sj-docx-1-dhj-10.1177_20552076231225133 - Supplemental material for Barriers and facilitators of health professionals in adopting digital health-related tools for medication appropriateness: A systematic review [file sj-docx-1-dhj-10.1177_20552076231225133.docx]

**Barriers and facilitators of health professionals in adopting digital health-related tools for medication appropriateness: a systematic review.**

Daniela A. Rodrigues, Maria Roque, Ramona Mateos-Campos, Adolfo Figueiras, Maria Teresa Herdeiro and Fátima Roque

**Supplementary Information (SI)**

**Table S1**: PRISMA 2020 Checklist

| **Section and Topic** | **Item #** | **Checklist item** | **Location where item is reported** |
| --- | --- | --- | --- |
| **TITLE** | | |  |
| Title | 1 | Identify the report as a systematic review. | Page 1 |
| **ABSTRACT** | | |  |
| Abstract | 2 | See the PRISMA 2020 for Abstracts checklist. | Page 1 |
| **INTRODUCTION** | | |  |
| Rationale | 3 | Describe the rationale for the review in the context of existing knowledge. | Pages 1-3 |
| Objectives | 4 | Provide an explicit statement of the objective(s) or question(s) the review addresses. | Page 3 |
| **METHODS** | | |  |
| Eligibility criteria | 5 | Specify the inclusion and exclusion criteria for the review and how studies were grouped for the syntheses. | Pages 3 and 4 |
| Information sources | 6 | Specify all databases, registers, websites, organisations, reference lists and other sources searched or consulted to identify studies. Specify the date when each source was last searched or consulted. | Page 3 |
| Search strategy | 7 | Present the full search strategies for all databases, registers and websites, including any filters and limits used. | Page 3 |
| Selection process | 8 | Specify the methods used to decide whether a study met the inclusion criteria of the review, including how many reviewers screened each record and each report retrieved, whether they worked independently, and if applicable, details of automation tools used in the process. | Pages 3 and 4 |
| Data collection process | 9 | Specify the methods used to collect data from reports, including how many reviewers collected data from each report, whether they worked independently, any processes for obtaining or confirming data from study investigators, and if applicable, details of automation tools used in the process. | Pages 4 and 5 |
| Data items | 10a | List and define all outcomes for which data were sought. Specify whether all results that were compatible with each outcome domain in each study were sought (e.g. for all measures, time points, analyses), and if not, the methods used to decide which results to collect. | Pages 4 and 5 |
|  | 10b | List and define all other variables for which data were sought (e.g. participant and intervention characteristics, funding sources). Describe any assumptions made about any missing or unclear information. | Pages 4 and 5 |
| Study risk of bias assessment | 11 | Specify the methods used to assess risk of bias in the included studies, including details of the tool(s) used, how many reviewers assessed each study and whether they worked independently, and if applicable, details of automation tools used in the process. | Pages 4 and 5 |
| Effect measures | 12 | Specify for each outcome the effect measure(s) (e.g. risk ratio, mean difference) used in the synthesis or presentation of results. | - |
| Synthesis methods | 13a | Describe the processes used to decide which studies were eligible for each synthesis (e.g. tabulating the study intervention characteristics and comparing against the planned groups for each synthesis (item #5)). | Pages 3 and 4 |
|  | 13b | Describe any methods required to prepare the data for presentation or synthesis, such as handling of missing summary statistics, or data conversions. | Page 5 |
|  | 13c | Describe any methods used to tabulate or visually display results of individual studies and syntheses. | Pages 4 and 5 |
|  | 13d | Describe any methods used to synthesize results and provide a rationale for the choice(s). If meta-analysis was performed, describe the model(s), method(s) to identify the presence and extent of statistical heterogeneity, and software package(s) used. | Pages 3-5 |
|  | 13e | Describe any methods used to explore possible causes of heterogeneity among study results (e.g. subgroup analysis, meta-regression). | - |
|  | 13f | Describe any sensitivity analyses conducted to assess robustness of the synthesized results. | - |
| Reporting bias assessment | 14 | Describe any methods used to assess risk of bias due to missing results in a synthesis (arising from reporting biases). | - |
| Certainty assessment | 15 | Describe any methods used to assess certainty (or confidence) in the body of evidence for an outcome. | - |
| **RESULTS** | | |  |
| Study selection | 16a | Describe the results of the search and selection process, from the number of records identified in the search to the number of studies included in the review, ideally using a flow diagram. | Pages 5 and 6 |
|  | 16b | Cite studies that might appear to meet the inclusion criteria, but which were excluded, and explain why they were excluded. | Page 5 |
| Study characteristics | 17 | Cite each included study and present its characteristics. | Pages 5 and 6 |
| Risk of bias in studies | 18 | Present assessments of risk of bias for each included study. | Page 7 and 15 |
| Results of individual studies | 19 | For all outcomes, present, for each study: (a) summary statistics for each group (where appropriate) and (b) an effect estimate and its precision (e.g. confidence/credible interval), ideally using structured tables or plots. | Table 1 |
| Results of syntheses | 20a | For each synthesis, briefly summarise the characteristics and risk of bias among contributing studies. | Page 7 and 15 |
|  | 20b | Present results of all statistical syntheses conducted. If meta-analysis was done, present for each the summary estimate and its precision (e.g. confidence/credible interval) and measures of statistical heterogeneity. If comparing groups, describe the direction of the effect. | Page 7-9 |
|  | 20c | Present results of all investigations of possible causes of heterogeneity among study results. | - |
|  | 20d | Present results of all sensitivity analyses conducted to assess the robustness of the synthesized results. | - |
| Reporting biases | 21 | Present assessments of risk of bias due to missing results (arising from reporting biases) for each synthesis assessed. | - |
| Certainty of evidence | 22 | Present assessments of certainty (or confidence) in the body of evidence for each outcome assessed. | - |
| **DISCUSSION** | | |  |
| Discussion | 23a | Provide a general interpretation of the results in the context of other evidence. | Pages 20-23 |
|  | 23b | Discuss any limitations of the evidence included in the review. | Page 23 |
|  | 23c | Discuss any limitations of the review processes used. | Page 23 |
|  | 23d | Discuss implications of the results for practice, policy, and future research. | Pages 20-24 |
| **OTHER INFORMATION** | | |  |
| Registration and protocol | 24a | Provide registration information for the review, including register name and registration number, or state that the review was not registered. | Page 3 |
|  | 24b | Indicate where the review protocol can be accessed, or state that a protocol was not prepared. | Page 3 |
|  | 24c | Describe and explain any amendments to information provided at registration or in the protocol. | - |
| Support | 25 | Describe sources of financial or non-financial support for the review, and the role of the funders or sponsors in the review. | Page 24 |
| Competing interests | 26 | Declare any competing interests of review authors. | Page 24 |
| Availability of data, code and other materials | 27 | Report which of the following are publicly available and where they can be found: template data collection forms; data extracted from included studies; data used for all analyses; analytic code; any other materials used in the review. | Supplementary material |

*From:*  Page MJ, McKenzie JE, Bossuyt PM, Boutron I, Hoffmann TC, Mulrow CD, et al. The PRISMA 2020 statement: an updated guideline for reporting systematic reviews. BMJ 2021;372:n71. doi: 10.1136/bmj.n71

**Table S2**: EMBASE and PubMed full search strategy.

| **EMBASE ALL<1 January 2000 to 28 October 2022>**  **Search date: 28 October 2022** | | |
| --- | --- | --- |
| # | **Searches** | **Results** |
| 1 | (barrier OR facilitator OR attitudes OR beliefs OR knowledge) AND (adopt OR adoption OR implementation*) AND (health professional OR health provider OR clinician OR physician OR GP OR general practitioner OR nurse OR pharmacist) AND (digital health related tool OR digital health tool OR mobile health OR mhealth OR m-health OR electronic health OR ehealth OR e-health OR telehealth OR clinical decision support system OR computerized clinical decision support system) AND (medication appropriateness OR drug appropriateness OR appropriate medication OR inappropriate medication OR inadequate prescription OR quality prescription OR adequate prescription) | 28 |
| **PubMed ALL<1 January 2000 to 28 October 2022>**  **Search date: 28 October 2022** | | |
| # | **Searches** | **Results** |
| 1 | (barrier OR facilitator OR attitudes OR beliefs OR knowledge) AND (adopt OR adoption OR implementation*) AND (health professional OR health provider OR clinician OR physician OR GP OR general practitioner OR nurse OR pharmacist) AND (digital health related tool OR digital health tool OR mobile health OR mhealth OR m-health OR electronic health OR ehealth OR e-health OR telehealth OR clinical decision support system OR computerized clinical decision support system) AND (medication appropriateness OR drug appropriateness OR appropriate medication OR inappropriate medication OR inadequate prescription OR quality prescription OR adequate prescription) | 1312 |

**Table S3**: Excluded articles with reason (n=66).

| No | Authors | Year | Title | Reason for exclusion |
| --- | --- | --- | --- | --- |
|  | Abuzaid et al | 2022 | Assessment of the Willingness of Radiologists and Radiographers to Accept the Integration of Artificial Intelligence into Radiology Practice | Do not address medication appropriateness |
|  | Aquino et al | 2022 | Patients' and Providers' Perspectives on and Needs of Telemonitoring to Support Clinical Management and Self-care of People at High Risk for Preeclampsia: Qualitative Study | Do not address medication appropriateness |
|  | Brünn et al | 2022 | Use of an Electronic Medication Management Support System in Patients with Polypharmacy in General Practice: A Quantitative Process Evaluation of the AdAM Trial | Do not present analysis of barriers or facilitators as an outcome |
|  | Kidd et al | 2022 | Improving assessment and progress monitoring in alcohol use disorder: An implementation evaluation of the instant assessment and personalised feedback system (iAx) | Do not address medication appropriateness |
|  | Liu et al | 2022 | Clinician Acceptance of Order Sets for Pain Management: A Survey in Two Urban Hospitals | Do not address medication appropriateness |
|  | Michiels-Corsten et al | 2022 | MediQuit - an electronic deprescribing tool: a pilot study in German primary care; GPs' and patients' perspectives | Do not present analysis of barriers or facilitators as an outcome |
|  | Moss et al | 2022 | Physicians' Views on Utilization of an Electronic Health Record-Embedded Calculator to Assess Risk for Venous Thromboembolism among Medical Inpatients: A Qualitative Study | Do not address medication appropriateness |
|  | Novakowski et al | 2022 | Health worker perspectives of Smart Triage, a digital triaging platform for quality improvement at a referral hospital in Uganda: a qualitative analysis | Do not address medication appropriateness |
|  | Regge et al | 2022 | Encouraging Digital Patient Portal Use in Ambulatory Surgery: A Mixed Method Research of Patients and Health Care Professionals Experiences and Perceptions | Do not address medication appropriateness |
|  | Soobiah et al | 2022 | Understanding Engagement and the Potential Impact of an Electronic Drug Repository: Multi-Methods Study | Do not address medication appropriateness |
|  | Sibbald et al | 2022 | Electronic Diagnostic Support in Emergency Physician Triage: Qualitative Study With Thematic Analysis of Interviews | Do not address medication appropriateness |
|  | Silvestri et al | 2022 | Desired Characteristics of a Clinical Decision Support System for Early Sepsis Recognition: Interview Study Among Hospital-Based Clinicians | Do not address medication appropriateness |
|  | Smith et al | 2022 | Exploring cancer patients', caregivers', and clinicians' utilisation and experiences of telehealth services during COVID-19: A qualitative study | Do not address medication appropriateness |
|  | Thield et al | 2022 | Adoption of a Postoperative Pain Self-Report Tool: Qualitative Study | Do not address medication appropriateness |
|  | Zaagsma et al | 2022 | 'It really is quite a different ballgame'. A qualitative study into the work experiences of remote support professionals | Do not address medication appropriateness |
|  | Zaslavsky et al | 2022 | Patient Digital Health Technologies to Support Primary Care Across Clinical Contexts: Survey of Primary Care Providers, Behavioral Health Consultants, and Nurses | Do not present analysis of barriers or facilitators as an outcome |
|  | Halwani et al | 2021 | Implementation of e-health innovative technologies in North Lebanon hospitals | Do not address medication appropriateness |
|  | Henkhaus et al | 2021 | Barriers and facilitators to use of a mobile HIV care model to re-engage and retain out-of-care people living with HIV in Atlanta, Georgia | Do not address medication appropriateness |
|  | Li et al | 2021 | Users' Perceptions Toward mHealth Technologies for Health and Well-being Monitoring in Pregnancy Care: Qualitative Interview Study | Do not address medication appropriateness |
|  | Muehlensiepen et al | 2021 | Opportunities and Barriers of Telemedicine in Rheumatology: A Participatory, Mixed-Methods Study | Do not address medication appropriateness |
|  | Nikolian et al | 2021 | A National Evaluation of Surgeon Experiences in Telemedicine for the Care of Hernia and Abdominal Core Health Patients | Do not address medication appropriateness |
|  | Pillay et al | 2021 | Doctor-perceived-barriers to telephone clinics at KwaZulu-Natal hospitals during the COVID-19 pandemic | Do not address medication appropriateness |
|  | Sarradon-Eck et al | 2021 | Attitudes of General Practitioners Toward Prescription of Mobile Health Apps: Qualitative Study | Do not address medication appropriateness |
|  | Taber et al | 2021 | Social dynamics of a population-level dashboard for antimicrobial stewardship: A qualitative analysis | Do not present analysis of barriers or facilitators as an outcome |
|  | Vilendrer et al | 2021 | An App-Based Intervention to Support First Responders and Essential Workers During the COVID-19 Pandemic: Needs Assessment and Mixed Methods Implementation Study | Do not address medication appropriateness |
|  | Ash et al | 2020 | Clinical Decision Support for Worker Health: A Five-Site Qualitative Needs Assessment in Primary Care Settings | Do not address medication appropriateness |
|  | Farokhzadian et al | 2020 | Nurses' experiences and viewpoints about the benefits of adopting information technology in health care: a qualitative study in Iran | Do not address medication appropriateness |
|  | Gui et al | 2020 | Physician champions' perspectives and practices on electronic health records implementation: challenges and strategies | Do not address medication appropriateness |
|  | Matsumoto et al | 2020 | Rheumatology Clinicians' Perceptions of Telerheumatology Within the Veterans Health Administration: A National Survey Study | Do not address medication appropriateness |
|  | Mulder-Wildemors et al | 2020 | Reducing Inappropriate Drug Use in Older Patients by Use of Clinical Decision Support in Community Pharmacy: A Mixed-Methods Evaluation | Studies that identify barriers and facilitators but did not associate them with the intention of healthcare professionals to adopt or use digital health-related tools |
|  | Kozikowski et al | 2019 | Care Team Perspectives and Acceptance of Telehealth in Scaling a Home-Based Primary Care Program: Qualitative Study | Do not address medication appropriateness |
|  | Morris et al | 2019 | Clinician Perspectives on mRehab Interventions and Technologies for People with Disabilities in the United States: A National Survey | Do not address medication appropriateness |
|  | Almutairi et al | 2018 | Physicians' Perceptions of Electronic Prescribing with Electronic Medical Records in Kuwaiti Primary Healthcare Centres | Do not address medication appropriateness |
|  | Johansson-Pajala et al | 2018 | Registered nurses' use of computerised decision support in medication reviews | Do not present analysis of barriers or facilitators as an outcome |
|  | Krog et al | 2018 | Barriers and facilitators to using a web-based tool for diagnosis and monitoring of patients with depression: a qualitative study among Danish general practitioners | Do not address medication appropriateness |
|  | Orengo-Aguayo et al | 2018 | Enhancing the Delivery of an Empirically-Supported Trauma-Focused Treatment for Adolescents: Providers' Views of the Role of Technology and Web-Based Resources | Do not address medication appropriateness |
|  | Beam et al | 2017 | Examining Perceptions of Computerized Physician Order Entry in a Neonatal Intensive Care Unit | Do not present analysis of barriers or facilitators as an outcome |
|  | Berkowitz et al | 2017 | Prescribing an App? Oncology Providers' Views on Mobile Health Apps for Cancer Care | Do not address medication appropriateness |
|  | DeMuro et al | 2017 | How Stakeholder Assessment of E-Prescribing Can Help Determine Incentives to Facilitate Management of Care: A Delphi Study | Do not present primary data |
|  | Mills et al | 2017 | Hospital staff views of prescribing and discharge communication before and after electronic prescribing system implementation | Do not present analysis of barriers or facilitators as an outcome |
|  | Morilla et al | 2017 | Implementing technology in healthcare: insights from physicians | Do not address medication appropriateness |
|  | Mozaffar et al | 2017 | Exploring the roots of unintended safety threats associated with the introduction of hospital ePrescribing systems and candidate avoidance and/or mitigation strategies: a qualitative study | Studies that identify barriers and facilitators but did not associate them with the intention of healthcare professionals to adopt or use digital health-related tools |
|  | Cresswell et al | 2016 | Establishing data-intensive healthcare: the case of Hospital Electronic Prescribing and Medicines Administration systems in Scotland | Do not address medication appropriateness |
|  | Peeters et al | 2016 | Use and Uptake of eHealth in General Practice: A Cross-Sectional Survey and Focus Group Study Among Health Care Users and General Practitioners | Do not address medication appropriateness |
|  | Rosenkrantz et al | 2016 | Technology-Assisted Virtual Consultation for Medical Imaging | Do not address medication appropriateness |
|  | Gangnon et al | 2015 | Connecting primary care clinics and community pharmacies through a nationwide electronic prescribing network: A qualitative study | Do not address medication appropriateness |
|  | Gider et al | 2015 | Evaluation of electronic prescription implications in Turkey: an investigation of the perceptions of physicians | Do not present analysis of barriers or facilitators as an outcome |
|  | Missiakos et al | 2015 | Identifying effective computerized strategies to prevent drug-drug interactions in hospital: A user-centered approach | Do not present analysis of barriers or facilitators as an outcome |
|  | Lakbala et al | 2014 | Physicians' perception and attitude toward electronic medical record | Do not address medication appropriateness |
|  | Levine et al | 2014 | Novel telemedicine technologies in geriatric chronic non-cancer pain: primary care providers' perspectives | Do not address medication appropriateness |
|  | Makam et al | 2013 | The good, the bad and the early adopters: providers' attitudes about a common, commercial EHR | Do not address medication appropriateness |
|  | Meulendijk et al | 2013 | General practitioners' attitudes towards decision-supported prescribing: an analysis of the Dutch primary care sector | Do not present analysis of barriers or facilitators as an outcome |
|  | Lehnbom et al | 2013 | A qualitative study of Swedes' opinions about shared electronic health records | Do not address medication appropriateness |
|  | Thomas et al | 2013 | Early experience with electronic prescribing of controlled substances in a community setting | Do not address medication appropriateness |
|  | Silow-Carroll et al | 2012 | Using electronic health records to improve quality and efficiency: the experiences of leading hospitals | Do not address medication appropriateness |
|  | Kim et al | 2011 | Attitude of korean primary care family physicians towards telehealth | Do not address medication appropriateness |
|  | Loscertales et al | 2011 | Facilitators in the implantation of telemedicine services. Perspective of professionals involved in its design and implementation | Do not address medication appropriateness |
|  | Paré et al | 2011 | Clinicians' perceptions of organizational readiness for change in the context of clinical information system projects: insights from two cross-sectional surveys | Do not address medication appropriateness |
|  | Hains et al | 2009 | Standardizing care in medical oncology: are Web-based systems the answer? | Do not address medication appropriateness |
|  | Kralewski et al | 2008 | Factors influencing physician use of clinical electronic information technologies after adoption by their medical group practices | Do not address medication appropriateness |
|  | Zaidi et al | 2008 | The role of perceptions of clinicians in their adoption of a web-based antibiotic approval system: do perceptions translate into actions? | Do not present analysis of barriers or facilitators as an outcome |
|  | Blaya et al | 2007 | A web-based laboratory information system to improve quality of care of tuberculosis patients in Peru: functional requirements, implementation and usage statistics | Do not address medication appropriateness |
|  | Grossman et al | 2007 | Physicians' experiences using commercial e-prescribing systems | Do not address medication appropriateness |
|  | Kramer et al | 2007 | Implementation of an electronic system for medication reconciliation | Do not present analysis of barriers or facilitators as an outcome |
|  | Chisolm et al | 2006 | The role of computerized order sets in pediatric inpatient asthma treatment | Do not present analysis of barriers or facilitators as an outcome |
|  | Barrett et al | 2005 | Physician perspectives on a pilot prescription monitoring program | Do not present analysis of barriers or facilitators as an outcome |

**Table S4:** Barriers and facilitators according to each study.

| Author (year) | Barriers | | | | | Facilitators | | | | |
| --- | --- | --- | --- | --- | --- | --- | --- | --- | --- | --- |
|  | **Technical** | **Organizational** | **Economical** | **User-related** | **Patient-related** | **Technical** | **Organizational** | **Economical** | **User-related** | **Patient-related** |
| Jeffries et al (2021) | SCSD  NT  AF | - | LFFI | NIDPR | - | EMS  EULHU  PS  APM  TSF  EAUTDI  ER | - | ICE | TS  EHPRP | - |
| Catho et al (2020) | AF  TC | MLIL | - | EUUDT  RC  LCA | - | EULHU  TSF  TP  RME  EAUTDI | - | ICE | TS | - |
| Jung et al (2020) | AF  LA  PUISD  LC  HU  NT  NCTU  BTU | - | - | LTGU | - | EULHU  IFW | - | - | CU  TS | - |
| Rieckert et al (2018) | UTU  LCOMS  EDS  TC | LI | LFFI | LT  DCDR  LTS  RDMPOS  FDCM | MPP  PCCM | UCK  UR  TSF  PS  FCA  IPI | - | - | TS  ACDR  EHPRP  ICC | EPMS |
| Koskela et al (2016) | PUISD  OMI  LVA  LPOR  AF  NCSPM  TC | LCIO | - | ODCAS  EUUDT | - | EULHU  EMS  ULMA  UR  BC  PS | - | - | - | - |
| Lugtenberg et al (2015) | NT  LVA  AF  PUISD  LSA  LC  LLCS  LCOMS  TC | - | - | LTS  NIDPR  LT  FMD | - | - | - | - | - | - |
| Charani et al (2013) | - | LI  LACOD  USDW | - | EUUDT  USDPP | - | UCK | - | - | IAP | - |
| Litvin et al (2012) | LVA  NT | LI  LACOD  AW | - | RC  DCDR | PMC | SMATSP  TP  TSF | NMCSAS  PAPW | - | CU  ACDR  IAPG | ICP  IPE |
| Vedel et al (2012) | TC | SST | - | EUUDT  NPU  NIDPR  DRLEBG  LCUS | - | EULHU | - | - | FST  ME  ST  CFU | IQC |
| Campion et al (2011) | LPR  PUISD  TC | DD  MLIL | - | - | - | - | - | - | TS | - |
| Hor et al (2010) | LASPS | LSPI | LFFI | LCA  FOS  LTS | - | HS  IPQ  RME | WIGRF |  | FST  CU |  |
| Weingart et al (2009) | HP  NIFS  AF  LSA  UTI | - | LFFI | LT  DNS | - | PPD  SPRP  RME  EULHU  ER  UR  PS | - | FI | IC | - |
| Varonen et al (2008) | LVA  PUISD | - | - | PEIDS  NIDPR  LCA  RC | - | FS  RBK  EULHU  UR  TP | - | AB | - | - |
| Schectman et al (2005) | NT | - | - | PFHWP | - | TSF  PS  EULHU | - | - | CU  ME | IQC  PA |
| Bury et al (2004) | NIFS  NT  ER | LACOD  MLIL | LFFI | DNS  LRCIP  PFPS | - | EULHU  SS  PPD | - | - | TS  EHPRP  IC | - |

**Table S5:** Most frequent reported barriers and facilitators according to the setting.

| *Primary care* | | *Hospital* | |
| --- | --- | --- | --- |
| Technical barriers | **Technical facilitators** | **Technical** **barriers** | **Technical facilitators** |
| AF  TC  NT | EULHU  TSF  OS | AF  TC  PUISD | EULHU |
| Organizational barriers | **Organizational facilitators** | **Organizational barriers** | **Organizational facilitators** |
| LI | - | MLIL | - |
| Economical barriers | **Economical facilitators** | **Economical barriers** | **Economical facilitators** |
| LFFI | - | - | - |
| User-related barriers | **User-related facilitators** | **User-related barriers** | **User-related facilitators** |
| NIDPR  LTS | TS  EHPRP  ACDR  CU | EUUDT | TS |
